# Draft Genome Sequence of Biocontrol Agent *Bacillus cereus* UW85

**DOI:** 10.1128/genomeA.00910-16

**Published:** 2016-09-01

**Authors:** Gabriel L. Lozano, Jonathan Holt, Jacques Ravel, David A. Rasko, Michael G. Thomas, Jo Handelsman

**Affiliations:** a Department of Molecular, Cellular and Developmental Biology, Yale University, New Haven, Connecticut, USA; b Department of Biological Sciences, Edgewood College, Madison, Wisconsin, USA; c Department of Microbiology and Immunology and Institute for Genome Sciences, University of Maryland School of Medicine, Baltimore, Maryland, USA; d Department of Bacteriology, University of Wisconsin-Madison, Madison, Wisconsin, USA

## Abstract

*Bacillus cereus* UW85 was isolated from a root of a field-grown alfalfa plant from Arlington, WI, and identified for its ability to suppress damping off, a disease caused by *Phytophthora megasperma* f. sp. *medicaginis* on alfalfa. Here, we report the draft genome sequence of *B. cereus* UW85, obtained by a combination of Sanger and Illumina sequencing.

## GENOME ANNOUNCEMENT

*Bacillus cereus* is a Gram-positive, ubiquitous spore-forming bacterium present in the soil, rhizosphere, and guts of several invertebrates (1). *B. cereus* UW85 was identified from 700 bacterial isolates from the roots of field-grown alfalfa plants. The collection was screened for the suppression of damping off caused by the oomycete *Phytophthora megasperma* f. sp. *medicaginis* on alfalfa seedlings (2). Damping off is characterized by browning of stem and root tissues, followed by girdling and seedling death. *B. cereus* UW85 produces two antibiotics, zwittermicin A (3) and kanosamine (4), which each exhibit broad-spectrum antimicrobial activity that contributes to the suppression of alfalfa seedling damping off, as demonstrated by analysis of mutants deficient in antibiotic production (4, 5). *B. cereus* UW85 can also protect tobacco seeds from infection by *Phytophthora parasitica* var. *nicotianae* (6) and cucumber fruit cotton leak, a disease caused by *Phytophthora aphanidermatum* (7). In the field, *B. cereus* UW85 enhances soybean nodulation (8) and can significantly increase the yield of several soybean cultivars (9). In addition, the presence of *B. cereus* UW85 on soybean seeds shapes the microbial community that develops subsequently in the rhizosphere (10).

The *B. cereus* UW85 genome was first sequenced using Sanger sequencing of a small-insert library (4 to 5 kb) and a large-insert library (10 to 12 kb), generating a total of ~51,000 reads, which were assembled using the Celera Assembler software (https://sourceforge.net/projects/wgs-assembler/) (11) into 271 contigs. Contigs were designated as originating from the chromosome or a plasmid with a BLAST comparison to the reference *B. cereus* isolates ATCC 10987 and ATCC 14579. Chromosomal contigs were ordered by Mauve (12) using the *B. cereus* ATCC 14579 genome (13) as a reference. A similar approach was used to assemble and order the plasmid contigs using several *B. cereus* group plasmids as references (14). Contigs were assembled manually by joining neighboring sequences with a linker sequence of unknown nucleotide characters labeled N. Gaps were filled using GapFiller (15) with 9,489,450 paired-end reads of 300 bp from an ~1-kb library generated on an Illumina MiSeq instrument, creating a merged assembly with both Sanger and Illumina data. The resulting assembled chromosome was 5,522,108 bp, consisting of 23 contigs, with an *N*_50_ contig size of 240,092 bp. Thirty-one contigs accounting for 881,969 bp, with an *N*_50_ contig size of 28,451 bp, showed greater similarity with *B. cereus* plasmid sequences. *B. cereus* is known to have an extremely varied plasmid profile, with strains carrying mixtures of plasmids ranging in size from 15 to 600 kb (16, 17). The zwittermicin A gene cluster (18, 19) is on one of the larger plasmid contigs, PC_11, which is ~150 kb. A second large plasmid, PC_30, is ~225 kb, has a region of similarity to the *Bacillus anthracis* pXO1 plasmid, and carries an uncharacterized gene cluster with similarity to a cluster encoding biosynthetic pathways for nonribosomal peptide synthesis machinery.

We anticipate that the genome sequence of *B. cereus* UW85, one of the best-characterized biocontrol agents, will facilitate discoveries about its plant growth-promoting activity, disease-suppressive properties, and potential for producing new antibiotics.

### Accession number(s).

This whole-genome shotgun project has been deposited at DDBJ/ENA/GenBank under the accession no. LYVD00000000. The version described in this paper is version LYVD01000000.
